# Psychosocial Impact of Cancer Care Disruptions in Women With Breast Cancer During the COVID-19 Pandemic

**DOI:** 10.3389/fpsyg.2021.662339

**Published:** 2021-06-14

**Authors:** Emily C. Soriano, Christine Perndorfer, Amy K. Otto, Alyssa L. Fenech, Scott D. Siegel, Diana Dickson-Witmer, Lydia Clements, Jean-Philippe Laurenceau

**Affiliations:** ^1^Department of Psychological and Brain Sciences, University of Delaware, Newark, DE, United States; ^2^Department of Public Health Sciences, University of Miami Miller School of Medicine, Miami, FL, United States; ^3^Christiana Care Health System, Helen F. Graham Cancer Center and Research Institute, Newark, DE, United States

**Keywords:** fear of cancer recurrence, fear of cancer progression, breast cancer, COVID-19, cancer survivorship

## Abstract

**Background:** The COVID-19 pandemic caused significant disruptions in cancer care, and preliminary research suggests that these disruptions are associated with increased levels of psychosocial distress among cancer survivors. The purpose of this study was to offer a descriptive report of the psychosocial functioning, perceived risk and fear of cancer progression, and COVID-19 pandemic impact and experiences in a unique, high-risk patient cohort: breast cancer survivors whose cancer treatment was delayed and/or changed due to the COVID-19 pandemic.

**Methods:** This cross-sectional study included 50 women with dual carcinoma *in situ*, lobular carcinoma *in situ*, or invasive breast cancer whose cancer surgery was postponed due to the pandemic. As they awaited delayed surgery or shortly after they received delayed surgery, participants completed questionnaires on psychosocial functioning (depression, anxiety, sleep, and quality of life), their perceived risk and fear of cancer progression, patient-provider communication about disruptions in their care, personal impact of the pandemic, worry/threat about COVID-19, and COVID-19 symptoms/diagnoses. Descriptive statistics and bivariate correlations were computed among continuous study variables. Independent samples *t*-tests explored group differences in psychosocial functioning between survivors who were still awaiting delayed surgery and those who had recently received it.

**Results:** Overall, the sample denied that the pandemic seriously negatively impacted their finances or resource access and reported low-to-moderate levels of psychosocial distress and fear about COVID-19. Twenty-six percent had clinically significant levels of fear of cancer progression, with levels comparable to other recent work. About a third were still awaiting delayed cancer surgery and this group reported lower satisfaction with communication from oncology providers but overall did not seem to report more psychosocial difficulties than those who already had surgery.

**Conclusion:** Shortly before or after primary breast cancer surgery that was delayed due to the COVID-19 pandemic, this sample of survivors appears to be generally managing well psychosocially. However, many psychosocial difficulties (e.g., fear of cancer recurrence/progression) typically have an onset after the completion of treatment, therefore, research should continue to follow this cohort of cancer survivors as the pandemic’s direct impact on their care likely increases their risk for these difficulties later in survivorship.

## Introduction

As of April 2021, there have been over 135 million COVID-19 cases and nearly 3 million deaths due to COVID-19 globally (WHO Coronavirus Disease (COVID-19) Dashboard, 2021). Individuals with cancer are at particularly elevated risk of a severe course of COVID-19 because they tend to be of older age (American Cancer Society, 2019) and are at a greater risk for needing intensive care and for mortality (Saini et al., 2020; Tian et al., 2020).

In the United States, the COVID-19 pandemic caused significant disruptions in non-COVID-related health care including cancer care. On March 13, 2020, the American College of Surgeons recommended that elective surgical procedures – including most cancer surgeries (John Hopkins Medicine, 2020) – be postponed to prevent COVID-19 transmission among health care providers and patients and mitigate the resource burden on the health care system (American College of Surgeons, 2020). In a survey on the impact of the COVID-19 pandemic on cancer survivors, 50% reported delays and/or changes in their health care (American Cancer Society Cancer Action Network, 2020). Among survivors with active cancer, 55% reported delays and/or changes in their health care, 13% reported not knowing when their care would be rescheduled, and 8% reported delays and/or changes in their anti-cancer therapy (American Cancer Society Cancer Action Network, 2020). Among breast cancer survivors specifically, 44% reported disruptions in their cancer care (Papautsky and Hamlish, 2020). Note that here, the definition of survivor is a person from the time of diagnosis to end-of-life, including those awaiting or actively receiving cancer treatment (we adopt this definition and use the terms survivor and patient interchangeably throughout this article; National Cancer Institute, 2011). Disruptions in breast cancer care included not only breast cancer surgery delays, but also delays and/or changes across the cancer care trajectory (e.g., diagnostic imaging and lab testing, anti-cancer therapies, and follow-up appointments; Papautsky and Hamlish, 2020). Given the great deal of uncertainty in pandemic cancer care and the potential impact of disruptions in care on cancer outcomes, cancer survivors diagnosed and treated during the COVID-19 pandemic may be at a particularly elevated risk for long-term psychosocial distress and poor mental health (Young et al., 2020).

Decisions to disrupt cancer care must carefully weigh the relative risks of COVID-19 exposure (and community spread) and poorer cancer prognosis due to care disruptions. Indeed, the impact of delays in breast cancer care on mortality has been well-documented (Hanna et al., 2020; Ho et al., 2020). The results from a review and meta-analysis indicated that just a 4-week delay in breast cancer surgery is associated with an 8% increase in the risk of death, after adjusting for important prognostic indicators including cancer stage (Hanna et al., 2020). The review concluded that if all individuals diagnosed with breast cancer in 1 year’s time were to experience a 12-week delay in surgery (e.g., due to a global pandemic), there would be an excess of 66,100 deaths in the United States alone (Hanna et al., 2020). A similar United Kingdom-based study predicted that these pandemic-related treatment delays will cause an 8–10% increase in breast cancer deaths in the first 5 years post-diagnosis (Maringe et al., 2020). Moreover, a study on excess mortality in individuals with cancer during the COVID-19 pandemic projected that there will be an excess of 33,890 deaths among this population in the United States as a result (Lai et al., 2020).

There have been numerous published guidelines to inform these complex decisions about cancer care during the COVID-19 pandemic (for reviews, see Garg et al., 2020; Zaniboni et al., 2020). For example, Smith et al. (2020) published recommendations for the prioritization of breast cancer surgeries delayed as a result of the pandemic. This system was developed using published data and the clinical judgment of a multidisciplinary breast oncology team. Per this system, each breast cancer survivor awaiting surgery is assigned a risk score based on patient and tumor factors, length of delay in cancer surgery, and for those who received neoadjuvant treatment, tumor response to this treatment. These scores form three classifications: (1) *Very urgent*, recommended the surgery in 2–4 weeks following the completion of chemotherapy; (2) *Limited delay acceptable*, recommended the surgery in 2–4 months (or longer if responding to neoadjuvant endocrine therapy); and (3) *Lowest priority*, recommended to wait until elective surgeries resume as usual. However, a recent review of the published guidelines for cancer care during the COVID-19 pandemic concluded that they are often discordant and based on limited evidence (Garg et al., 2020).

In addition to changes in health care systems and their decisions on prioritization, changes in functioning at the individual (person) level may also contribute to disruptions in cancer care during the COVID-19 pandemic. Breast cancer survivors who were diagnosed during the COVID-19 pandemic were found to refuse surgery at a higher rate than those diagnosed pre-pandemic, primarily due to fear of COVID-19 infection (Vanni et al., 2020). It seems those cancer survivors’ psychological and behavioral reactions to the pandemic (e.g., COVID-19-related fear) may be contributing to disruptions in care above and beyond those caused by system-level changes. Furthermore, COVID-19-related fear may be particularly high in this population. In a survey conducted with cancer survivors, caregivers, and healthcare workers early in the pandemic (i.e., April 2020), 66% of cancer survivors reported feeling “very much” or “extremely” fearful of COVID-19 – a rate significantly higher than that observed in healthcare workers (Ng et al., 2020). Compared to caregivers and health care workers, cancer survivors perceived themselves to be at greater risk for severe complications due to and non-recovery from COVID-19 (Ng et al., 2020). Evidence suggests that COVID-19-related fear may be highest among individuals with breast cancer as compared to individuals diagnosed with other cancers (the authors speculated that this may be due to gender differences; Sigorski et al., 2020).

In addition to uncontrollable system changes in their cancer care and their own psychological and behavioral responses to the pandemic, social distancing – strongly recommended for cancer survivors due to their high risk of COVID-19 infection – further increases this group’s risk of psychosocial difficulties. This includes loneliness and isolation (Garutti et al., 2020), which are among the most potent psychosocial influences on mental and physical health (Hawkley and Cacioppo, 2010). Indeed, loneliness was a top concern among cancer survivors seeking psycho-oncology treatment during the COVID-19 pandemic (Schellekens and Lee, 2020). Among individuals being treated for breast cancer during the pandemic, one in two reported moderate or severe levels of loneliness (Bargon et al., 2021).

In addition to the high rates of psychological distress in the general population during the COVID-19 pandemic (Xiong et al., 2020), an emerging body of work has examined the psychosocial impact of the COVID-19 pandemic on cancer survivors. This emerging evidence suggests that during the COVID-19 pandemic, individuals with cancer experience higher levels of depression and anxiety than the general population as well as cancer survivors pre-pandemic (Chen et al., 2020; Han et al., 2020; Ng et al., 2020; Wang et al., 2020). Research conducted in the epicenter of the pandemic in China suggests that levels of depression and anxiety may be comparable among breast cancer survivors and frontline female nurses and that breast cancer survivors had even higher levels of insomnia (Cui et al., 2020).

Moreover, emerging evidence also suggests that disruptions in cancer care are related to these mental health symptoms (Chen et al., 2020; Swainston et al., 2020); breast cancer survivors who reported the discontinuation of their cancer treatment due to the pandemic were more likely to report moderate or severe symptoms of depression, anxiety, and insomnia (Juanjuan et al., 2020). In addition, cancer survivors whose cancer care has been impacted by the pandemic may be at risk for experiencing more fear of cancer progression (FCP), which is the “fear, worry, or concern relating to the possibility that cancer will come back or progress,” (Lebel et al., 2016, p. 3266). Given the impact of cancer care disruptions on cancer outcomes, including mortality, FCP is a particularly relevant outcome for individuals diagnosed with cancer during the COVID-19 pandemic and whose cancer care was delayed or changed. Indeed, among breast cancer survivors, approximately 54% reported concerns regarding the efficacy of anti-cancer therapies that were delayed and/or changed due to the pandemic (Juanjuan et al., 2020). We are aware of two published studies examining FCP among cancer survivors during the COVID-19 pandemic (Chen et al., 2020; Massicotte et al., 2021). Among cancer survivors in the pandemic epicenter in China, 86.5% reported some degree of FCP, and importantly, the study found that having had disruptions in cancer care was significantly predictive of FCP (Chen et al., 2020). Another study conducted in Canada similarly found that among women with non-metastatic breast cancer receiving chemotherapy, 52.8% had clinically significant levels of FCP, but whether or not participants experienced treatment delays and/or changes due to the COVID-19 pandemic was not reported (Massicotte et al., 2021).

The primary aim of the present paper was to provide a comprehensive baseline characterization of the psychosocial functioning of a cohort of breast cancer survivors whose cancer care had been delayed and/or changed due to the COVID-19 pandemic in the United States. While prior studies examined psychosocial functioning among breast cancer survivors across the continuum of cancer care, little is known about the cohort whose surgery was delayed and/or changed due to the pandemic. Therefore, we conducted a cross-sectional study of survivors who were awaiting delayed surgery or who recently underwent delayed surgery. Our goal was to provide a broad description of a sample from this unique cohort who may be at high risk of experiencing long-term psychosocial sequalae as a result of the COVID-19 pandemic.

## Materials and Methods

### Participants

Data were from a cross-sectional study titled *Impact of COVID-19 on Women Recently Diagnosed with Breast Cancer* (Christiana Care Health System IRB approval: FWA00006557; CCC# 40079). The purpose of this study was to examine the psychosocial impact of the COVID-19 pandemic on women diagnosed with dual carcinoma *in situ* (DCIS), lobular carcinoma *in situ* (LCIS), or invasive breast cancer whose cancer surgery was postponed as a result of the pandemic. Following from their surgery status, all potential participants had non-metastatic (operable) breast cancer (Stage 0 to III). Eligibility for the study included women who (1) were diagnosed with DCIS, LCIS, or invasive breast cancer, (2) whose cancer surgery was postponed as a result of the COVID-19 pandemic, and (3) spoke English. Note that although LCIS is neither technically considered cancer nor “pre-cancer,” participants with DCIS/LCIS whose treatment plans included surgical intervention (i.e., excisional biopsy) to reduce the likelihood of disease progression and rule out any other disease process were included in this study. This decision was made based on the study’s focus on concerns about disease progression, the levels of which have been found in prior research to be comparable across patients DCIS/LCIS and those with stage I cancer (Liu et al., 2011).

Christiana Care Health System postponed all elective surgical procedures on March 17, 2020. As a result, 172 breast surgeries were postponed at Christiana Care’s Helen F. Graham Cancer Center and Research Institute. Breast surgeries resumed at the Cancer Center on May 15, 2020. To prioritize the scheduling of the large backlog of breast surgeries, the Helen F. Graham Cancer Center and Research Institute used a combination of the recommendations made by the COVID-19 pandemic breast cancer consortium and the system created by Smith et al. (Dietz et al., 2020; Smith et al., 2020). Each pending breast surgery case was assigned a risk score based on patient and tumor factors (e.g., age, tumor grade, and size), the length of delay in cancer surgery (e.g., time since biopsy), and for those who received neoadjuvant treatment, tumor response to this treatment (e.g., imaging response score and physical exam response score). Higher scores reflected greater potential risk and, therefore, greater urgency for surgery. There were some changes to the specific protocol and scoring procedure as new data emerged during the pandemic, and as a result, about half of potential participants did not have documented risk scores using the system created by Smith et al. (2020).

**Figure 1:**
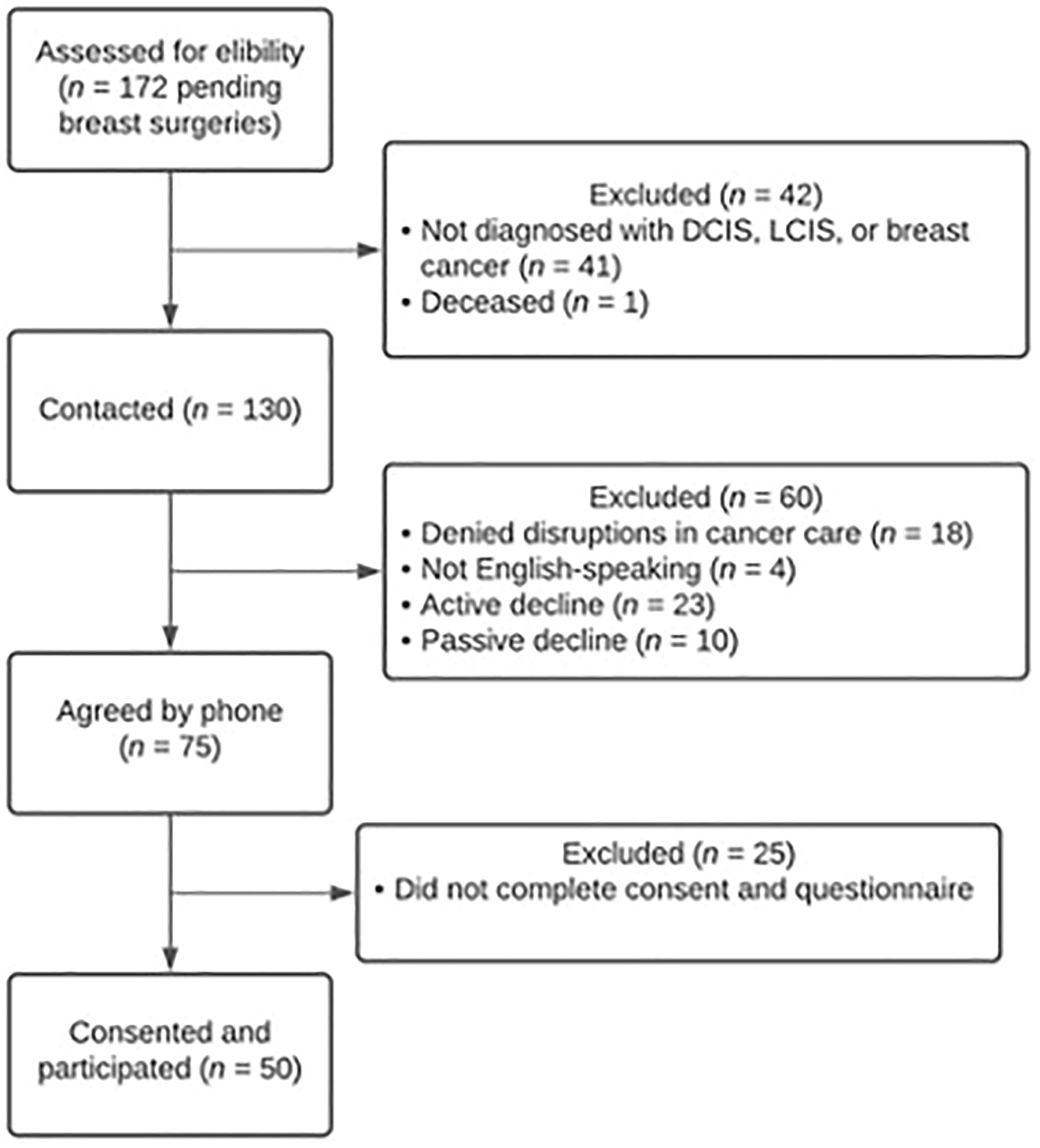
Participant flow.

Participant flow is detailed in Figure 1. Of the 172 cases pending for breast surgery, 41 had a diagnosis other than DCIS, LCIS, or invasive breast cancer (e.g., atypical ductal hyperplasia) and one was deceased by the start of the study. Of those contacted to participate (*n* = 130), 18 denied postponements in their cancer surgery, four did not speak English, 23 actively declined, and 10 passively declined. Seventy-five agreed by phone to participate and 50 completed the informed consent and cross-sectional survey.

As previously mentioned, breast surgeries at the Helen F. Graham Cancer Center and Research Institute resumed on May 15, 2020. Data collection for the present study occurred between June 27 and August 13, 2020. Therefore, by the time of data collection, most of the sample had received delayed breast cancer surgery (more detail described in section Results).

### Procedure

A list of the pending breast surgery cases was maintained by the Helen F. Graham Cancer Center and Research Institute and was used to screen for potential eligibility. All cases with a diagnosis of DCIS, LCIS, or invasive breast cancer (*n* = 130) were mailed a letter containing an invitation to participate, a brief description of the study, and study contact information. Approximately 1 week after mailing this letter, all potential participants were contacted by phone by a clinical psychology doctoral student. During these phone calls, a script was used to invite potential participants to take part in the study, describe the study procedures, describe the potential risks and benefits of participation, and answer potential participants’ questions about the study. Five potential participants indicated that they did not have access to the internet and were therefore offered to complete the informed consent form and study questionnaire *via* mail. Those who could not be reached were contacted by phone up to two additional times (for a maximum of three phone calls) before being considered as passive decliners.

Those who agreed by phone to participate (*n* = 75) were sent additional information about the study, the informed consent form, and study questionnaire. Those who expressed interest in completing the informed consent and study questionnaire online were sent these materials *via* email (*n* = 70) and those who expressed interest in completing the informed consent and study questionnaire *via* mail (*n* = 5) were sent these materials *via* mail. Those who were sent study materials *via* email received up to three reminder emails and one reminder call (spaced approximately 4 days apart) if they had not yet completed the informed consent and study questionnaire online. Those who were sent the study materials *via* email were not provided with reminder emails or calls. Forty-nine participants completed the informed consent (signed and dated electronic form) and study questionnaire online and one participant completed the informed consent and study questionnaire *via* mail on paper, resulting in a total sample of 50 participants. Participants were not compensated for taking part in the study.

The informed consent form included an authorization for the request of medical information. This authorization was optional and if authorized, allowed the research staff to access participants’ electronic medical records – specifically, medical oncology notes for more detailed clinical data such as breast cancer stage.

### Materials

See the summary of measures administered in Table 1.

**Table 1:** Measures administered.

Variable/measure	Description
Sociodemographic characteristics	Race, ethnicity, gender, education, and income
Cancer history and treatment	Prior cancer diagnoses and current cancer treatment
Patient-provider communication	Two items; content and perceived quality of communication re: COVID-19
COVID-19 impact	Five items; financial and resource access changes (Conway et al., 2020)
COVID-19-specific threat sensitivity	Three items; worry and perceived COVID-19 threat (Conway et al., 2020)
Cancer progression risk perception	Three items; perceived risk given COVID-19-related treatment changes
Fear of cancer progression (FCP)	Eight items; adapted from FCRI-SF (Fardell et al., 2017)
Generalized anxiety and depression	PROMIS Short Form Anxiety 4a and Depression 4a (Cella et al., 2010)
Sleep quality	One item from PSQI (Buysse et al., 1989)
Quality of life	One item from FACT-G (Cella et al., 1993)

Measures without a citation listed were developed by the authors for the purposes of this study. FCRI-SF = Fear of Cancer Recurrence Inventory-Short Form (Simard and Savard, 2009; Fardell et al., 2017); PSQI = Pittsburgh Sleep Quality Index (Buysse et al., 1989); FACT-G = Functional Assessment of Cancer Therapy-General (Cella et al., 1993).

#### Sociodemographic Characteristics

Participants reported on their race, ethnicity, gender identity, education, family income, pre-pandemic employment status, and COVID-19-related changes in employment status.

#### Cancer History and Treatment

Participants reported on the previous history of breast and other cancers. They were also were asked whether they had received surgery for their current breast cancer diagnosis, as of the day of questionnaire completion. If they responded *no*, they were asked whether their surgery had been scheduled. Finally, participants were asked whether they received neoadjuvant hormone therapy and/or chemotherapy.

#### Patient-Provider Communication About Pandemic-Related Treatment Changes

Two questions assessed survivors’ perceptions of communication with their health care team about delays and/or changes in cancer treatment due to the pandemic. The first question asked what, if anything, the oncologist told the survivor about how the delay/change in treatment might affect the risk of cancer progression; response options were, “*They told me my risk would be **lower** because of the delay/changes in my treatment*,” “*They told me my risk would be **about the same***,” “*They told me my risk would be **higher** because of the delay/changes in my treatment*,” and “*They **did not talk to me** about how the delay/changes might affect my risk*.” The second question assessed survivor satisfaction with the communication from their medical team about COVID-19-related delays/changes in their cancer treatment. Response options ranged from one (“*not at all satisfied*”) to five (“*completely satisfied*”).

#### COVID-19 Impact

COVID-19 impact was assessed using a modified version of the Coronavirus Impacts Questionnaire-Short Version (Conway et al., 2020). The Coronavirus Impacts Questionnaire-Short Version was modified by replacing one resource impact item [“*It has been difficult for me to get the things I need due to the coronavirus (COVID-19)*”] with an item assessing impact to health insurance coverage specifically [“*My health insurance coverage has been negatively affected by the coronavirus (COVID-19)*”]. Because psychosocial functioning had already been assessed in this study, two psychosocial impact items [“*I have become depressed because of the coronavirus (COVID-19)*” and “*The coronavirus outbreak has impacted my psychological health negatively*”] were replaced with one item assessing impact to household responsibilities [“*My household responsibilities (child care, chores) have increased and/or are more difficult to manage due to the coronavirus (COVID-19)*”]. Each item was rated from one (“*not true of me at all*”) to seven (“*very true of me*”). Items were averaged to assess the overall COVID-19 impact, and the modified scale in this sample had acceptable reliability (α = 0.81).

#### COVID-19 Experiences

Participants responded to the Personal Diagnoses/Symptoms Scale of the Coronavirus Experiences Questionnaire-Short Version (Conway et al., 2020), which includes three items assessing whether participants had ever been diagnosed with COVID-19, had COVID-19-like symptoms at any point in the prior 2 months, or had been sick with something other than COVID-19 and breast cancer in the prior 2 months.

#### COVID-19-Specific Threat Sensitivity

COVID-19-specific threat sensitivity was assessed using a modified version of the Perceived Coronavirus Threat Questionnaire-Short Version (Conway et al., 2020), which consists of three items assessing how worried or threatened respondents feel about COVID-19. Given that individuals with cancer are at increased risk for severe complications from COVID-19, we added a fourth item that read, “*I am anxious or worried about surviving the coronavirus (COVID-19) if I caught it*.” All four items were rated from one (“*not true of me at all*”) to seven (“*very true of me*”). Responses were averaged to create a composite score, with greater scores reflecting greater COVID-19-specific threat sensitivity. The alpha coefficient (*α* = 0.91) reflected acceptable reliability.

#### Cancer Progression Risk Perception

Three items measured the perceived risk of cancer progression. One item assessed overall concern about cancer progression [“*How concerned are you about your cancer progressing (growing or spreading in the same or another part of the body)?*”] with responses ranging from one (“*not at all concerned*”) to seven (“*extremely concerned*”). The second item assessed the perceived risk of progression (“*Considering any delays or changes in your cancer treatment due to coronavirus (COVID-19), what do you think of your chance is of your cancer progressing?*”), with a continuous slider scale ranging from 0% (“*no chance of cancer progression*”) to 100% (“*cancer will definitely progress*”). The final item assessed the perceived change (“*much lower*,” “*about the same*,” or “*much higher*”) in progression risk due to COVID-19-related delays/changes in treatment [“*Considering information from your medical team, overall, how do you think your chance of cancer progression compares to breast cancer patients whose treatment was not delayed or changed due to coronavirus (COVID-19)?*”].

#### Fear of Cancer Progression

FCP was assessed using a modified version of the Fear of Cancer Recurrence Inventory Severity subscale (Simard and Savard, 2009), also termed as FCRI-Short Form (FCRI-SF; Fardell et al., 2017). The FCRI is a well-validated measure of FCP but was specifically designed to assess recurrence rather than progression, the former being more relevant for survivors who have completed cancer treatment and the latter for survivors with active cancer (i.e., the current sample). The FCRI-SF consists of nine items assessing intrusive thoughts about and the perceived risk of recurrence over the past month. Eight items were modified to refer to *progression* instead of *recurrence* (e.g., “*I was worried or anxious about the possibility of cancer recurrence*” became “*I was worried or anxious about the possibility of cancer progression*”) and one item was dropped (“*How long have you been thinking about the possibility of cancer recurrence?*”) because some of the response options (e.g., “*A few years*” and “*Several years*”) were not applicable, as only recently diagnosed survivors participated. All eight items were rated from zero to four. Responses were averaged to create a composite score, with higher scores reflecting greater FCP. The alpha coefficient (*α* = 0.88) reflected acceptable reliability of this modified scale. The current recommended cutoff score to establish clinical levels on the FCRI-SF is a sum score > 22 (Fardell et al., 2017), equivalent to a mean score > 2.44, which we used here as an approximate indicator of FCP severity on our adapted measure. To facilitate comparison to other recently published findings on FCP during the COVID-19 pandemic (Massicotte et al., 2021), we also reported the percentage of scores exceeding the lower cutoff of > 13 (equivalent to a mean score > 1.44), which is often still used as recommended by original measure developers (Simard and Savard, 2009).

#### Generalized Anxiety and Depressive Symptoms

The PROMIS Short Form Anxiety 4a and Depression 4a were administered as brief measures of generalized anxiety and depressive symptoms (Cella et al., 2010). Each scale includes four items assessing the severity of symptoms experienced in the past 7 days, with responses ranging from one (“*never*”) to five (“*always*”). A composite score for each scale is converted to a *T*-score (population *M* = 50, SD = 10). For these PROMIS scales, *T*-scores between 55 and 60 are considered mild, 60–70 moderate, and > 70 severe. Reliability was acceptable for the Anxiety (*α* = 0.93) and Depression scales (*α* = 0.89).

#### Sleep Quality

A single item from the Pittsburgh Sleep Quality Index (PSQI; Buysse et al., 1989) asked participants, “*During the past month, how would you rate your sleep quality overall?*” Responses ranged from one (“*very good*”) to four (“*very bad*”).

#### Quality of Life

A single item from the Functional Assessment of Cancer Therapy Functional Well Being subscale (FACT-G; Cella et al., 1993) was used to assess subjective quality of life-based on “*how you have been feeling the past 7 days*.” The item was “*I am content with the quality of my life right now*,” with responses ranging from one (“*not at all*”) to five (“*very much*”).

### Statistical Analysis

Analyses were conducted using SPSS v.26 (IBM Corp., 2019). Most variables had no missing values, with the exception of a few skipped questions [e.g., two participants skipped the question about the perceived risk of cancer progression (%)]. Descriptive analyses were based on all available data from the full sample of 50 participants. Descriptive sample statistics, including means, frequencies, standard deviations, and ranges were computed for all variables, and their distributions examined. Bivariate correlations were also computed among all key study variables. Independent samples *t*-tests were conducted to explore group differences in psychosocial functioning between survivors who were still awaiting delayed surgery (*n* = 17) and those who had recently received it (*n* = 33). Values of *p* (*α* = 0.05) for these tests were reported but interpreted cautiously with a greater focus on effect sizes given the relatively small sample size (*N* = 50).

## Results

### Medical Record Data

A list of the pending breast surgery cases was maintained by the Helen F. Graham Cancer Center and Research Institute. This list included data on diagnosis (e.g., DCIS, LCIS, or invasive breast cancer) but not breast cancer stage for those with invasive breast cancer. Per this list, most women in the final sample of 50 survivors were diagnosed with invasive breast cancer (78%), with 18% having been diagnosed with DCIS and 4% with LCIS. Forty-one women (82%) authorized access to their medical records. Of these 41 women, most (*n* = 18, 36%) had been diagnosed with clinical stage I breast cancer. Ten women (20%) had been diagnosed with clinical stage 0, 7 women (14%) with clinical stage II, and 4 women (8%) with stage III. Breast cancer stage could not be obtained for two women who authorized access to their medical records (e.g., participant decided to seek treatment out of state). As mentioned in the Participants section above, less than half of the current sample (44%) had documented risk scores calculated using the system created by Smith et al. (Smith et al., 2020; the remaining were missing because of changes in the site’s internal risk scoring protocol early in the COVID-19 pandemic). The mean risk score for these participants was 17.5, which falls in the (2) *Limited delay acceptable* group (score between 10 and 29; *n* = 15 had scores in this range), and Smith et al.’s recommendation was that this group can generally wait 2–4 months or longer if they continued to respond to neoadjuvant treatment. Only three participants had scores that fell in the (3) *Lowest priority* group (score < 10), where the recommendation was that this group can likely wait until elective surgeries resume. Only four participants would be in the (1) *Very urgent* group (score ≥ 30), where surgery is recommended in 2–4 weeks.

### Psychosocial Characteristics

Descriptive statistics for self-report variables are shown in Table 2.

**Table 2:** Descriptive statistics.

Variable	Mean	SD	Range
COVID-19 impact	2.23	1.48	1–7
COVID-19 threat sensitivity	4.14	1.82	1–7
Perceived risk of progression (concern)	3.94	2.00	1–7
Perceived risk of progression (0–100%)	30%	28%	0–100%
Fear of progression	1.85	0.89	0.38–3.71
PROMIS anxiety	54.71	9.67	40.30–81.60
PROMIS depression	48.99	7.90	41.00–73.30
Sleep quality	2.24	0.77	1–4
Quality of life	3.42	1.18	1–5

*N* = 50 for all variables except for the perceived risk of progression (0–100%), an item that two participants skipped (*n* = 48). Only continuous variables included in table.

#### Sociodemographic Characteristics

All participants identified as women. The average age of participants was 60.1 years (SD = 13.2). The majority of the sample identified as White (74%), 20% Black or African-American, and 4% Asian (one participant skipped the question). Most participants identified as not Hispanic/Latino (92%), with one participant who skipped this item. Fifty-eight percent of participants reported having a college or post-graduate degree. The modal annual family income exceeded $100,000. Prior to the pandemic, 52% of participants indicated that they were employed full-time for wages, 28% retired, 8% self-employed, 4% employed part-time for wages, 4% out of work for a year or more, 4% unable to work (disabled), and 2% were homemakers. Over a third (36%) of participants reported that there had been no changes in their work because of COVID-19, 26% reported that they transitioned to working from home, 6% reported an *increase* in work responsibilities, 6% reported a *decrease* in work responsibilities, 6% reported being essential workers with regular physical presence required, 4% reported decreased pay, and 1% reported being laid off, fired, or forced to close business (multiple response options were allowed for this item).

#### Cancer History and Treatment

The majority indicated that this was their first breast cancer diagnosis (88%) and denied having any other cancer diagnoses in the past (84%). At the time of data collection (between June 27 and August 13, 2020), most of the sample (66%) self-reported that they had already received surgery for their current breast cancer. Of those who indicated they did not yet have surgery (34%), 41% reported that they had a scheduled surgery date in the future while the remaining 59% did not. Regarding neoadjuvant treatment, a portion (74%) of this sample reported that they received hormone or chemotherapy treatment prior to surgery.

#### Patient-Provider Communication About Pandemic-Related Treatment Changes

Regarding communication from their oncology team, 48% reported that the impact of treatment delays on their cancer progression risk was not discussed at all, whereas 44% reported being told that their risk would be *about the same*. Only 4% reported being told that their risk was *higher* because of the treatment delay/changes, and 4% reported being told that their risk was *lower* because of the treatment delay/changes. Regarding satisfaction with this communication from their health care providers, the modal response was “*very satisfied*,” with less than 15% of the sample reporting poor to low (“*not at all*” or “*a little*”) satisfaction (*M* = 3.92, SD = 1.11).

#### COVID-19 Impact

The overall COVID-19 impact scores were relatively low on average (*M* = 2.23, see Table 2). Responses to the five items assessing COVID-19 impact on personal finances and access to essential resources, including health care, indicated that at least half the sample denied experiencing any of the impact areas assessed, with a modal score of 1 (the lowest possible impact) on each item. Less than 20% of participants had scores greater than the scale midpoint on any of the impact items. The mean response to the item assessing whether COVID-19 had negative financial impacts was 2.83 (SD = 2.17), with a small subgroup endorsing moderate to extreme negative impacts (20%). Even fewer participants endorsed great negative impact in the form of household responsibilities (*M* = 2.59, SD = 2.24), job income loss (*M* = 2.00, SD = 1.99), access to essential resources (*M* = 2.32, SD = 2.00), and health insurance changes (*M* = 1.45, SD = 1.50).

#### COVID-19 Experiences

None of the participants reported having received a COVID-19 diagnosis. Only five participants (10%) said that they had COVID-19-like symptoms at some point over the past 2 months, and only two (4%) said they had been sick with something other than COVID-19 and breast cancer over that same period.

#### COVID-19-Specific Threat Sensitivity

There was substantial spread in participant scores on the COVID-19 threat sensitivity items (*M* = 4.14, SD = 1.82, range = 1–7). The composite scores were approximately normally distributed, although most scores were in the moderate range, many were also observed at both the extreme low and high ends of the scale.

#### Cancer Progression Risk Perception

There was also marked variability in participants’ self-reported concern about cancer progression, *M* = 3.94, SD = 2.00, range = 1–7. The modal response was two on a scale of one (“*not at all concerned*”) to seven (“*extremely concerned*”). Forty percent of participants reported concern in the low-moderate range (score < 4) and 20% reported concern at the high end of the scale (score > 6). In light of any COVID-19-related delays or changes in care, participants’ own estimate of their risk of cancer progression on average was 30% (SD = 28%). A third (33%) of the sample reported that their chance of cancer progression was 12% or less and 33% reported estimates between 15 and 30%. Eight percent of participants indicated a 50% risk of progression, with the remaining responses scattered between 30 and 100%. The majority of participants (80%) endorsed the belief that their risk of progression was “*about the same*” compared to patients whose treatment was unaffected by COVID-19, with only 12% stating they felt their risk was much higher and 8% felt their risk was much lower.

#### Fear of Cancer Progression

FCP composite scores were approximately normally distributed in this sample, *M* = 1.85, SD = 0.89, range = 0.38–3.71. Using the recommended clinical cutoff of 2.44 on the original FCRI-SF as a rough point of comparison (Fardell et al., 2017), FCP appeared moderate on average. About a quarer (26%) of the sample had scores exceeding this cutoff, suggesting elevated FCP of potential clinical concern. Using the lower clinical cutoff of 1.44 (recommended by Simard and Savard, 2009 but later found to be too low for optimal sensitivity and specificity; Fardell et al., 2017), 60% of participants had scores in the clinically significant range.

#### Generalized Anxiety and Depressive Symptoms

The mean PROMIS Anxiety *T*-score was 54.71, SD = 9.67, with 64% of participants having scores greater than 50 (population mean). Twenty-six percent of participants had anxiety scores in the mild range, 26% in the moderate range, and 4% in the severe range. The mean PROMIS Depression *T*-score was 48.99, SD = 7.90, with 44% of participants having scores over 50. Rates of clinical depressive symptoms were relatively low in this sample, with 14% reporting mild symptoms, 6% reporting moderate, and 2% severe.

#### Sleep quality

The modal response to the question concerning subjective sleep quality over the past month was “*fairly good*” (score = 2), *M* = 2.24, SD = 0.77, range = 1–4. Only three participants reported “very bad” sleep quality.

#### Quality of life

Survey responses indicated that on average, participants were generally content with their quality of life, *M* = 3.42, SD = 1.18, range = 1–5, with the modal response being “*somewhat content*” (score = 3). About 15% of participants indicated a low quality of life (score < 3).

### Bivariate Correlations

Bivariate correlations among continuous variables are shown in Table 3. We found that greater patient-provider communication around pandemic-related surgery delays was significantly correlated with lower COVID-19 impact (*r* = −0.50, *p* < 0.01), lower perceived risk of cancer progression (*r* = −0.32, *p* < 0.05), lower FCP (*r* = −0.36, *p* < 0.05), and fewer depression symptoms (*r* = −0.29, *p* < 0.05). We also found that higher COVID-19 impact scores significantly correlated with greater perceived risk of cancer progression (*r* = 0.29, *p* < 0.05), more generalized anxiety symptoms (*r* = 0.35, *p* < 0.05), and lower quality of life (*r* = −0.39, *p* < 0.01). Higher sensitivity to the threat of COVID-19 was significantly correlated with generalized anxiety levels (*r* = 0.44, *p* < 0.01), depression symptoms (*r* = 0.34, *p* < 0.05), and poorer sleep (*r* = 0.29, *p* < 0.05), but not with concern about or the perceived risk of cancer progression. Perceived risk (concern), the perceived risk estimate (0–100%), and FCP were all highly inter-related (*r*s.64–0.69, *p*s < 0.01). Of these three variables, FCP showed the highest number of significant bivariate relationships with other psychosocial variables – including communication satisfaction, perceived risk (concern), perceived risk (0–100%), generalized anxiety, depression, sleep quality, and quality of life – all suggesting evidence of poorer psychosocial functioning (see Table 3 for full results).

**Table 3:** Bivariate correlations.

	1	2	3	4	5	6	7	8	9
1. Communication satisfaction	-								
2. COVID-19 impact	−0.501^∗∗^	-							
3. COVID-19 threat sensitivity	−0.138	0.144	-						
4. Perceived risk (concern)	−0.186	0.165	0.109	-					
5. Perceived risk (0–100%)	−0.319^∗^	0.288^∗^	−0.079	0.641^∗∗^	-				
6. Fear of cancer progression	−0.359^∗^	0.274^†^	0.260^†^	0.689^∗∗^	0.659^∗∗^	-			
7. PROMIS anxiety	−0.217	0.350^∗^	0.441^∗∗^	0.514^∗∗^	0.319^∗^	0.681^∗∗^	-		
8. PROMIS depression	−0.293^∗^	0.199	0.343^∗^	0.463^∗∗^	0.222	0.614^∗∗^	0.679^∗∗^	-	
9. Sleep quality^a^	−0.082	0.199	0.289^∗^	0.287^∗^	0.254^†^	0.374^∗∗^	0.382^∗∗^	0.330^∗^	-
10. Quality of life	0.219	−0.391^∗∗^	−0.211	−0.412^∗∗^	−0.399^∗∗^	−0.413^∗∗^	−0.550^∗∗^	−0.407^∗∗^	−0.562^∗∗^

*N* = 50.

∗∗ *p* < 0.01;

∗ *p* < 0.05.

† *p* < 0.10.

a Higher score indicate worse sleep quality.

### Mean Differences by Surgery Status

Independent samples *t*-tests examined mean differences in continuous variables between participants who already received surgery and those still awaiting their surgery date. *T*-statistics were used to compute effect sizes (Hedges’ *g*). Results are shown in Table 4. A moderate-to-large-sized effect (*g* = 0.721) was observed for mean differences in communication satisfaction [*t*(48) = −2.42, *p* = 0.019], such that survivors who had already received their postponed breast cancer surgery were more satisfied with the communication from their oncology providers (*M* = 3.88) than those still awaiting their postponed surgery (*M* = 3.12). A moderate-sized effect (*g* = 0.56) was also observed for a mean difference in PROMIS Depression scores [*t*(48) = 1.87, *p* = 0.067], indicating that survivors still awaiting surgery had somewhat higher levels of depressive symptoms (*M* = 52) than those post-surgery (*M* = 48). A moderate-sized effect (*g* = 0.53) was also found for the perceived risk of cancer progression (0–100%, *t*(46) = 1.73, *p* = 0.091), such that survivors awaiting surgery also estimated that they had higher risks of cancer progression (*M* = 40%) than those post-surgery (*M* = 25%). The remaining *t*-tests revealed smaller mean differences (*g* < 0.4) between the groups (i.e., weak evidence found for meaningful group differences in levels of COVID-19 impact, COVID-19 threat sensitivity, concern about the perceived risk of progression, FCP, PROMIS Anxiety, sleep quality, and quality of life).

**Table 4:** Means of continuous variables by cancer surgery status.

	Mean (SD)		*t*(48)	*p*	Effect size (Hedges’ *g*)^a^
	Awaiting surgery (*n* = 17)	Received surgery (*n* = 33)			
Communication satisfaction	3.12 (1.17)	3.88 (0.99)	−2.42^∗^	0.019	0.721
COVID-19 impact	2.62 (1.79)	2.03 (1.28)	1.36	0.181	0.401
COVID-19 threat sensitivity	4.25 (2.00)	4.08 (1.75)	0.30	0.762	0.093
Perceived risk (concern)	4.41 (2.27)	3.70 (1.85)	1.20	0.236	0.355
Perceived risk (0–100%)^✣^	39.56 (32.01)	24.94 (25.33)	1.73^†^	0.091	0.528
Fear of cancer progression	2.05 (0.99)	1.75 (0.84)	1.12	0.267	0.336
PROMIS anxiety	55.00 (8.51)	54.55 (10.34)	0.15	0.879	0.046
PROMIS depression	51.83 (6.89)	47.52 (8.08)	1.87^†^	0.067	0.559
Sleep quality	2.47 (0.72)	2.12 (0.78)	1.54	0.130	0.460
Quality of life	3.18 (1.42)	3.55 (1.03)	−1.05	0.299	0.315

*N* = 50.

∗ *p* < 0.05.

† *p* < 0.10.

✣ df = 46 (two participants skipped this question).

a Hedges’ *g* is in pooled standard deviation units (similar to Cohen’s *d*) and accounts for unequal sample sizes.

## Discussion

This study aimed to provide a comprehensive description of the psychosocial functioning of a unique cohort, high-risk of breast cancer survivors in the United States whose cancer care had been delayed and/or changed due to the COVID-19 pandemic. These are survivors who had been recently diagnosed with non-metastatic, operable breast cancer and thus likely have a favorable prognosis, with a 5-year relative survival rate of 99% for localized breast cancer and 86% for regional breast cancer (American Cancer Society, Inc., 2021). Prior to the pandemic, a large body of literature documents lingering psychosocial concerns among cancer survivors, such as fear of cancer recurrence, that can be problematic well into the years after cancer has been successfully treated (Stanton, 2006). However, the current sample represents a group who may be at an even greater risk of experiencing these and other difficulties given that their cancer treatment was directly impacted (i.e., delayed and/or changed) due to the COVID-19 pandemic. In this paper, we described how this group fared psychosocially shortly before or after their postponed breast cancer surgery.

About two-thirds of the sample had recently received their breast cancer surgery, which had been delayed due to the COVID-19 pandemic, whereas the remaining third comprised survivors still awaiting their delayed surgery date. The majority of these survivors had received neoadjuvant treatment (either endocrine therapy or chemotherapy), which may have been recommended to mitigate increased risks associated with delays in primary surgery. In terms of race and ethnicity, the sample is roughly comparable to the typical cancer survivor in this Mid-Atlantic region of the United States (US Census Bureau, 2019). However, the current sample was less representative in terms of socioeconomic status (higher income and education level). This higher socioeconomic status is also consistent with the finding that the majority of participants denied that the COVID-19 pandemic seriously impacted their finances or limited their access to essential resources, including health care. In addition, few reported job losses or pay cuts during the pandemic. There was a small subgroup of participants who reported job loss or pay decreases (5%) or reported being frontline essential workers (6%). On the other hand, 20% of the current sample was African-American, a group disproportionately impacted by COVID-19 (Tai et al., 2020). These sample characteristics are important to consider when interpreting the results discussed below.

Despite the fact that this sample was selected on the basis of their breast cancer treatment being delayed and/or changed due to the pandemic, 48% reported that their oncology providers never discussed with them how this disruption would affect their cancer prognosis (i.e., risk of cancer progression). Most of the remaining participants (44%) reported that they were told the delay and/or change in their treatment would not change their prognosis or cancer progression risk. Because we do not have detailed medical records available for these participants, the accuracy of these statements cannot be estimated. Consistent with the relatively high proportion of participants who denied having a discussion with their providers about this, 18 survivors identified by the hospital records as having delayed and/or changed breast cancer surgery declined to participate because they did not believe that their cancer treatment was altered due to the pandemic. Unfortunately, we did not collect corroborating data from participants’ oncology providers to determine the extent to which these results reflect actual (objective) patient-provider interactions, participants’ comprehension of information communicated by providers, and/or other individual-level factors that may color their perception, memory, or judgment about prior discussions with their providers. Future research may be able to explore these questions by incorporating more detailed clinical data, data from patients’ oncology providers, and/or direct observation of patient-provider discussions about risk and prognosis. Nonetheless, it may have been challenging for oncology providers to navigate these discussions with survivors due to the unprecedented nature of the pandemic and limited empirical data on change in risk due to these delays. Importantly, despite this, most participants reported satisfaction with their communication by their oncology team. Although, interestingly and perhaps understandably, satisfaction was higher on average among those who already had surgery and lower among those still awaiting their delayed surgery.

There also was a moderate-sized correlation between satisfaction with oncology provider communication and low COVID-19 impact. Sociodemographic factors may partially explain this effect; for example, individuals who are Black or living in poverty are both more likely to be affected by COVID-19 (CDC, 2020) and less likely to be satisfied with provider communication and their medical care in general (compared to Whites or higher-income individuals, e.g., Haviland et al., 2005; McFarland et al., 2017). In addition, results of the current study showed that those who were more satisfied with communication tended to report lower estimates of their perceived risk of cancer progression and, correspondingly, lower FCP. It is possible that receiving or perceiving “better news” from a provider (i.e., being told that they have a lower risk of cancer progression) causes the patient to feel more satisfied with the provider’s communication and also serves to lower the patient’s own risk estimate and, consequently, FCP.

We also examined several indicators of psychosocial distress, including the perceived threat of COVID-19, FCP, generalized anxiety and depressive symptoms, sleep quality, and perceived quality of life. The pattern of findings for these variables suggested that overall, this sample on average reported low-to-moderate levels of psychosocial concerns and fear related to COVID-19. Given that extant data are suggestive of potentially poorer cancer prognosis when surgery is delayed (as was the case for these participants), we speculated that this sample is likely at higher risk for experiencing high FCP, which is already a relatively normative experience among cancer survivors even in the absence of a global pandemic. Results indicated that about a quarter of this sample experienced clinically elevated FCP, as defined by the most recent psychometric evidence (Fardell et al., 2017). A recent study of non-metastatic breast cancer survivors during the COVID-19 pandemic found that 53% had FCP scores in the clinical range (Massicotte et al., 2021), but used a lower cutoff score recommended in earlier work (Simard and Savard, 2009). Using this same lower cutoff, 60% of the current sample had clinical levels of FCP, yielding findings consistent with those reported by Massicotte et al. (2021).

Perhaps surprisingly, mean levels of FCP did not significantly differ between survivors pre‐ vs. post-surgery, yet prior work found delays in cancer care to be significantly related to survivors’ FCP (Chen et al., 2020). Critically, however, FCP is known to become prevalent and potentially problematic among cancer survivors after treatment ends and their cancer has been successfully treated (King et al., 2000; McKinley, 2000). Therefore, it will be of key importance that future work continues to follow this cohort of cancer survivors as they progress through this survivorship trajectory – re-assessing them after they have completed surgery and adjuvant treatment, when told by their providers that their cancer is in remission. Anecdotally, during this time in the survivorship trajectory individuals begin to have questions, doubts, and fears about their cancer coming back. Indeed, it seems reasonable to speculate that this cohort is still at greater risk for experiencing clinically significant FCP (or fear of recurrence) than their pre-pandemic counterparts. Moreover, it is possible that future research will reveal additional areas of difficulty for survivors whose treatment was delayed and/or changed as a result of the pandemic.

This study had a number of strengths. Most notably, this was the first study, to our knowledge, to specifically target the assessment of breast cancer survivors whose primary cancer surgery was postponed due to the COVID-19 pandemic. Prior research on cancer survivors during the pandemic has not selected participants on the basis of their care being directly affected (i.e., delayed). At the same time, our sample was heterogeneous with regard to being pre‐ vs. post-surgery, receipt of neoadjuvant treatment, and sociodemographic variables. Nevertheless, there were also important limitations to this study. First, the sample size was relatively small and thus these results should be considered as tentative pending a well-powered replication. Second, while this sample was racially and ethnically representative of the patient population, participants were generally financially secure, highly educated, and did not report being severely impacted by the COVID-19 pandemic. Thus, more research is needed to better understand the needs of even higher risk groups of cancer survivors during and after the COVID-19 pandemic. Future research could benefit from identifying and describing other patient populations whose cancer care was affected by COVID-19, particularly patients who may be at higher risk, including those of lower socioeconomic status, as well as patients with other cancer diagnoses; this work may help to identify key sociodemographic or clinical characteristics that impact psychosocial response to COVID-19. Third and finally, the use of short forms or single items to assess psychological symptoms and multidimensional constructs may not fully capture these concepts, thus limiting the interpretation of the results.

## Data Availability Statement

The raw data supporting the conclusions of this article will be made available by the authors, without undue reservation.

## Ethics Statement

The studies involving human participants were reviewed and approved by Christiana Care Health System IRB approval: FWA00006557; CCC# 40079. The patients/participants provided their written informed consent to participate in this study.

## Author Contributions

ES: conceptualization, data curation, formal analysis, methodology, and writing – original draft. CP: conceptualization, data curation, formal analysis, investigation, methodology, project administration, and writing – original draft. AO: conceptualization, methodology, and writing – review and editing. AF: writing – review and editing. SS: conceptualization, methodology, resources, and supervision, writing – review and editing. DD-W: conceptualization, resources, supervision, and writing – review and editing. LC: conceptualization, resources, supervision, writing – review and editing. J-PL: conceptualization, data curation, resources, supervision, and writing – review and editing. All authors contributed to the article and approved the submitted version.

### Conflict of Interest

The authors declare that the research was conducted in the absence of any commercial or financial relationships that could be construed as a potential conflict of interest.
